# Short-term outcomes in the upper airway with tooth-bone-borne vs bone-borne rapid maxillary expanders

**DOI:** 10.1186/s12903-023-03461-6

**Published:** 2023-10-04

**Authors:** Javier Echarri-Nicolás, María José González-Olmo, Pablo Echarri-Labiondo, Martin Romero

**Affiliations:** 1 Doctoral Program in Health Sciences, International PhD School, Rey Juan Carlos University (URJC), Madrid, Spain; 2 Department of Orthodontics, University Rey Juan Carlos, Avda de Atenas S/N 28922, Alcorcón, Madrid Spain; 3 Athenea Dental Institute, San Jorge University, Zaragoza, Spain

**Keywords:** Cone-beam computed tomography (CBCT), Microimplant assisted rapid palatal expansion (MARPE), Bone expansion, Upper airway, Maxillary transverse deficiency, Miniscrew-Assisted Rapid Palatal Expansion (MARPE), Bone-Anchored Maxillary Expander (BAME)

## Abstract

**Background:**

This study compared the area and minimal section of the nasal cavity, nasopharynx, oropharynx, and hypopharynx in cases treated with different methods of microimplant-assisted expansion.

**Methods:**

Based on a pilot study to calculate the sample size, 30 patients with transverse maxillary deficiency over 14 years of age were retrospectively selected. These patients had received two different types of microimplant-assisted maxillary expansion treatment (MARPE and BAME). The patient underwent Cone-Beam computed tomography (CBCT) before and after treatment (mean time 1.5 months) with MARPE or BAME and upper airway measurements (volume and minimum cross-sectional area) were taken to assess upper airways changes and compare changes between the groups. A paired sample t-test was performed to evaluate the T0-T1 change of airway measurements obtained with MARPE and BAME, and a student t-test to compare changes in airway measurements between MARPE and BAME.

**Results:**

This investigation shows a statistically significant increase in total nasopharyngeal airway volume (0.59 ± 1.42 cm3; *p* < 0.01), total oropharyngeal airway volume (3.83 ± 7.53 cm^3^; *p* < 0.01) and minimum oropharyngeal cross-section (53.23 ± 126.46 mm^2^; *p* < 0.05) in all cases treated with micro-screw assisted expansion. The minimal cross-sectional area of the oropharynx ((79.12 ± 142.28 mm2; *p* < 0.05) and hypopharynx (59.87 ± 89.79 mm^2^; *p* < 0.05) showed significant changes for cases treated with BAME. As for the comparison between cases treated with MARPE and BAME, no differences in upper airway changes have been observed, except for the minimum cross-sectional area of the nasal cavity, which increases for MARPE (52.05 ± 132.91 mm2) and decreases for BAME (-34.10 ± 90.85 mm2).

**Conclusions:**

A significant increase in total area and minimal section at the level of nasopharynx and oropharynx was observed in cases treated with BAME. Regarding the comparison of MARPE and BAME treatments, no differences were found in the total airway volume and minimal section in upper airway except for the minimum cross section of the nasal cavity that increases for MARPE and decreases for BAME.

**Supplementary Information:**

The online version contains supplementary material available at 10.1186/s12903-023-03461-6.

## Background

Posterior crossbite is a malocclusion present in 5% of the world’s population with permanent dentition [1, 2]. Rapid palatal expansion (RPE) in adults causes a purely orthodontic expansion. Dental effects include increased labiolingual angulation of the molars [3, 4]. This limitation is due to the mid-palatal suture ossification, which makes the separation of hemimaxillary portions impossible with tooth-supported separators [3]. The effects of RPE in the upper airway in young patients [5–9] and adult patients [10, 11] have also been described.

The classic approach to posterior crossbite in adults has been Surgically Assisted Rapid Palatal Expansion (SARPE) [12–15]. This approach involves carrying out a surgical separation of the already ossified mid-palatal suture. By means of an intraoral expansion device, the skeletal transverse dimension can be increased.

The Microimplant-Assisted Rapid Palatal Expansion (MARPE) technique has been widely described in the literature [16–22] and is characterized by a reduction of the excessive load exerted by conventional appliances on the labial periodontal ligament of the teeth used as anchorage. It consists of a tooth-bone borne device with 2–4 bicortical microscrews from the palatal cortical bone to the nasal floor as a retention, placed in the posterior area of the palate, regardless of the age or sex of the patient. Bone-Anchored Maxillary Expansion (BAME) therapy has recently been described for the cases in which less dental effect is desired [23, 24]. With this therapy the activation forces are directed directly to the basal bone.

Rapid palatal expansion has been reported to confer significant benefits to patients’ airway [5–8, 10–12, 22, 25]. In particular, MARPE has been shown to increase the transverse dimension and volume of the upper airway [5, 6, 8, 10, 23], increased nasal width [5, 26, 27], improved airflow [8, 24, 28] and decreased respiratory resistance [24, 27]. Such effects have been reported in adults that underwent SARPE [11]. However, there is a dearth of information pertaining to different MARPE therapies; Bazzani et al. [29] compared skeletal changes in patients treated with MARPE and BAME with microimplants that were placed in different ways.

This investigation had two primary objectives. One was to analyze and compare the area and minimal section of the nasal cavity, nasopharynx, oropharynx, and hypopharynx for different bone-separation therapies. The second was to compare the area and minimal section of the nasal cavity, nasopharynx, oropharynx, and hypopharynx for MARPE- and BAME-type separation devices.

Our findings provide orthodontists with important information about expansion therapies that could most benefit patients’ upper airways.

## Materials and methods

### Design and participants

**Fig. 1:**
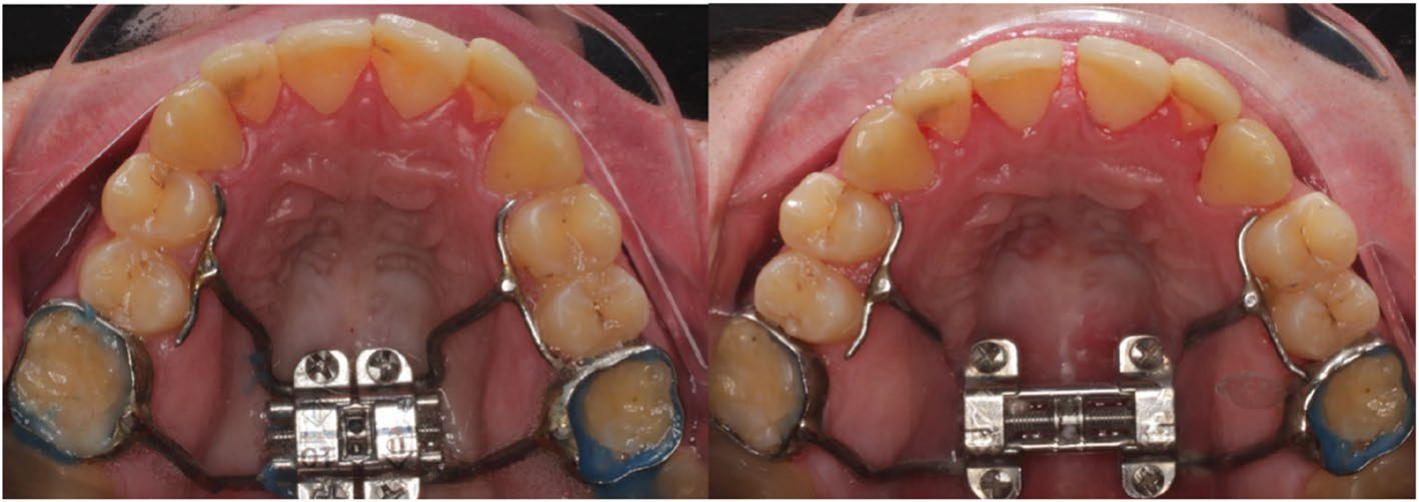
Pre and postexpansion MARPE device design

**Fig. 2:**
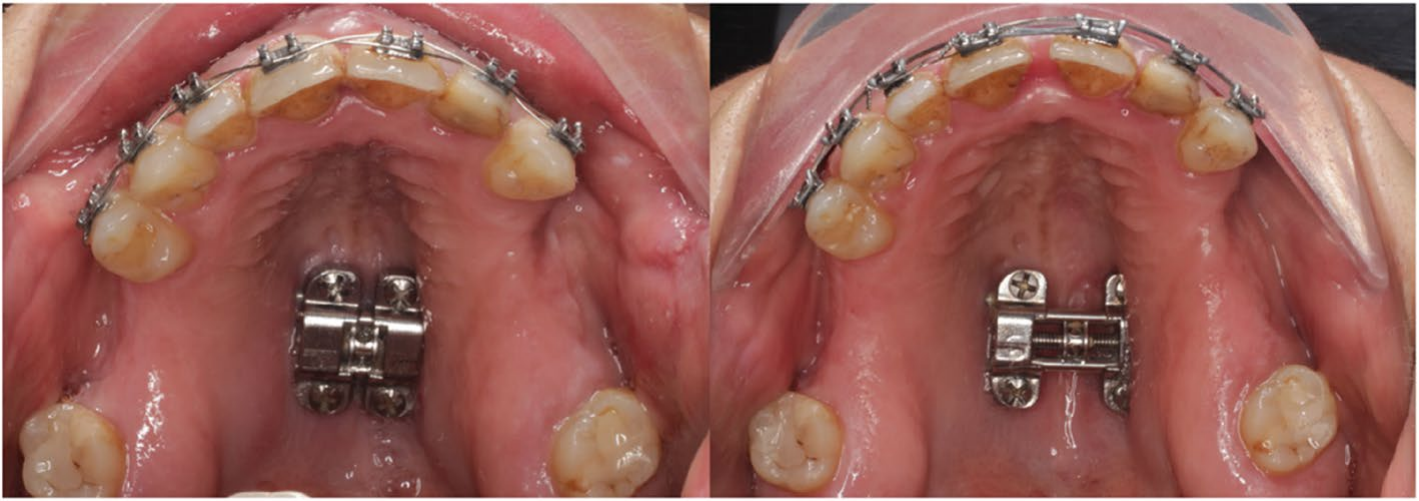
Pre and postexpansion BAME device design

Patient data were analyzed retrospectively from September 2021 to September 2022. Data were exploited from September 2022 to February 2023. These patients were undergoing treatment with MARPE or BAME in a private dental clinic (Clinic Athenea Dental Institute). MARPE technique consists of a tooth-bone-borne appliance with a retention using four bicortical microscrews from the palatal cortical bone to the nasal floor (Fig. 1). In 2013, BAME concept described by Winsauer et al. [23] was introduced (Fig. 2). It is an expansion screw attached to four or six microimplants in the palatal area without tooth support.

The inclusion criteria of the study were patients with maxillary compression without counter-indications for surgery and who had undergone microimplant-assisted maxillary expansion treatment. The exclusion criteria from the study were patients with craniofacial malformations, patients with fissured palate. All patients were informed of the orthodontic procedure with the potential risks and benefits, and an informed consent was obtained to undergo treatment and be included in the study. The age of inclusion was set at 14 years of age or older, since it is considered more complicated to perform a conventional separation after that age. These are the subjects in which a microimplant-assisted expansion is indicated [30–32]. Demographics and sample images were used and the information was anonymized. The study protocol was reviewed and approved by the Ethics Committee of the Rey Juan Carlos University, with internal number (1504202110721).

The sample size was calculated using Jamovi 2.3.18 assuming a study power of 80% and an alpha error of 5%. As there were no previous studies comparing dimensional changes in the upper airway comparing BAME and MARPE, sample size calculations were based on the results of a pilot study performed in ten patients. The calculated mean ± standard deviation (SD) of the total nasopharyngeal volume change of BAME and MARPE was 1.33 cm^3^ ± 2.6 and 0.05 cm^3^ ± 1.3, respectively. Based on comparison of means, using two-tailed test, it was calculated that accepting an alpha risk of 0.1 and a beta risk of 0.2 in a bilateral contrast, 13 subjects per group are required to detect a difference equal to or greater than 1.28 cm^3^ units. The common standard deviation is assumed to be 1.3 cm^3^. This was eventually increased to 15 per group, bringing the total sample size required to 30 patients.

### Procedure and measurements

Palalign® Round Head Type microimplants (Osteonic Co. Ltd., Seoul, Republic of Korea), made of Ti6Al4V alloy, with a 1.8 mm diameter and lengths of 10, 12, 14, or 16 mm, depending on the case, were used to ensure bicortical fixation, thus increasing stability and reducing the risk of microimplant deformation and fracture [29]. All devices were digitally designed, and the placement of microscrews was guided digitally to minimize clinical placement errors [33]. The Power MARPE Type 1 screw (Osteonic Co. Ltd., Seoul, Republic of Korea) was used, with an activation rate of 4 turns per day until the interincisal diastema appeared, and then 2 turns per day until the overcorrection of 1.5 mm per side was achieved. All treatments were performed by the same orthodontist.

The patient was subjected to a CT-type radiographic recording (NewTom Giano HR with 300 μm voxel size and a 16 × 18 cm FOV) before and after MARPE or BAME treatment, and the following indicators were calculated on that 3D X-ray before (T0) and after treatment (T1) to confirm the midpalatal suture opening to avoid the surgery. The mean time between measurements was 1.5 months.

**Fig. 3:**
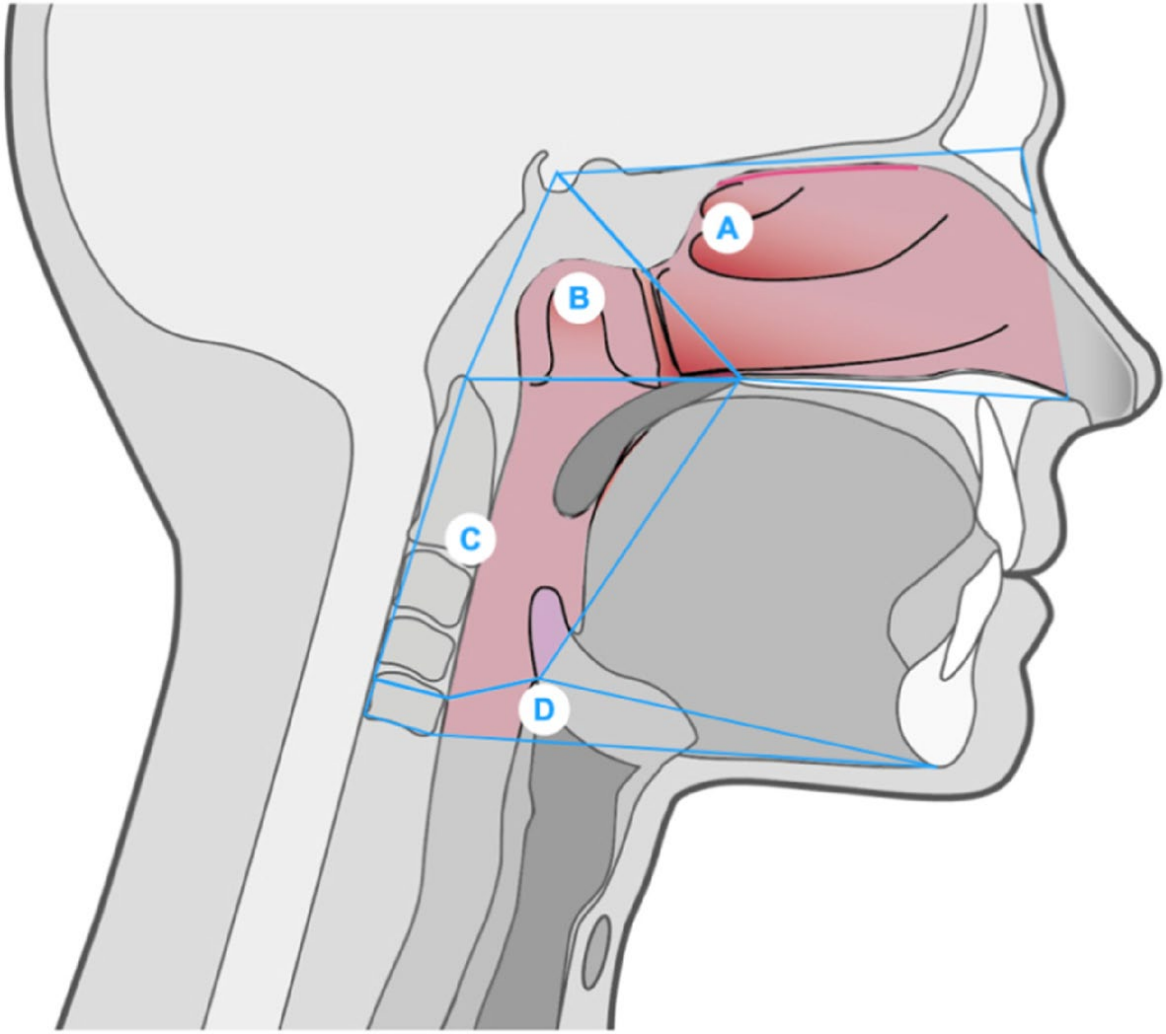
Anatomical regions of the upper airway: (**A**) Nasal cavity, (**B**) Nasopharynx, (**C**) Oropharynx, (**D**) Hypopharynx

For the study of the upper airway, it is anatomically divided (Fig. 3) according to the tomographic description of Smith et al. [26] The division is performed in the nasal cavity, nasopharynx, oropharynx and hypopharynx in the Cone-Beam Computed Tomography (CBCT) (Table 1). It is not acceptable to use 2D radiography because of the overlapping structures described in the literature [28].

**Table 1:** Parameters evaluated in the study

Measurements	Description
Nasal Cavity Total Airway Volume (NCTAV)	Anterior limit: Line connecting the anterior nasal spine (ANS) with the tip of the nasal bone Posterior limit: Line extending from Sella to the posterior nasal spine (PNS) Upper limit: Line connecting Nasion (N) and Sella (S) Lower limit: Line extending from the ANS to the PNS
Nasal Cavity Minimal Cross-section (NCMCS)	
Nasopharynx Total Airway Volume (NPTAV)	Anterior limit: Line extending from S to PNS Posterior limit: Line extending from S to the tip of the odontoid process of the atlas vertebra Lower limit: Line extending from PNS to the tip of the odontoid process of the atlas vertebra
Nasopharynx Minimal Cross-section (NPMCS)	
Oropharynx Total Airway Volume (OPTAV)	Anterior limit: Line extending from PNS to the base of the epiglottis Posterior limit: Line extending from the tip of the odontoid process to the superior-posterior edge of the CV4 Upper limit: Line extending from the PNS to the tip of the odontoid process Lower limit: Line extending from the base of the epiglottis to the supero-posterior edge of the CV4
Oropharynx Minimal Cross-section (OPMCS)	
Hypopharynx Total Airway Volume (HPTAV)	Anterior limit: Line extending from the base of the epiglottis to the lower edge of the symphysis Posterior limit: Line extending from the upper-posterior edge of CV4 to the lower-posterior edge of CV4 Upper limit: Line that extends from the base of the epiglottis to the upper-posterior edge of the CV4 Lower limit: Line extending from the lower-posterior edge of CV4 to the inferior edge of the symphysis
Hypopharynx Minimal Cross-section (HPMCS)	

### Statistical analysis

All statistical analyses were performed using the Statistical Package for the Social Sciences version 28.0 for Windows (SPSS, IBM, Armonk, NY). A descriptive analysis was carried out to expose the details of the sample such as age, sex, type of appliance and airway measurements. Three measurements were also carried out for each indicator and for each investigation time, and the Measurement Error (ME) was calculated. Measurements were repeated after two weeks to assess intraexaminer reliability using the intraclass correlation coefficient (ICC). The ICC was calculated considering ICC < 0.4 as low, between 0.4 and 0.75 as acceptable and > 0.75 as high. The Shapiro-Wilks test is performed to check the normality of the variables. Finding a non-significant *p*-value and therefore establishing parametric tests. Subsequently, a paired sample t-test was performed to evaluate the T0-T1 change of airway measurements obtained with MARPE and BAME, and a student t-test to compare changes in airway measurements between MARPE and BAME. In addition, Cohen's d was used for the effect of the sample in the analysis of the differences of the means with the t-test. A measure with low effect was considered d ≈ 0.2, medium d ≈ 0.5 and high d ≈ 0.8 [34].

## Results

### General descriptive analysis

Thirty patients were included in our analysis. The MARPE technique was used in 15 patients (50%), and the BAME technique was used in 15 patients (50%). Thirteen of the patients were men and 17 were women. The mean age of the cohort was 21.8 ± 6.0 years. The youngest patient was 14 years old, and the oldest patient was 34 years old.

Microimplant-Assisted Rapid Palatal Expansion therapy was used in 6 of the male subjects, and BAME was used in the remaining 7. Microimplant-Assisted Rapid Palatal Expansion therapy was used in 9 of the female subjects, and BAME was used in the remaining 6. There were no differences in gender distribution (X (1) = 0.136; *p* = 0.713). We evaluated the age-related characteristics of the two groups at T0 and found no significant differences (MARPE group: 20.53 ± 5.74 years; BAME group: 23.07 ± 6.21 years; t = 1.16, *p* = 0.256).

We also evaluated airway differences at T0: NCTAV (*p* = 0.879); NCMCS (*p* = 0.493); NPTAV (*p* = 0.623); NPMCS (*p* = 0.123); OPTAV (*p* = 0.523); OPMCS (*p* = 0.600); HPTAV (*p* = 0.154); HPMCS (*p* = 0.606). No statistically significant differences were found between the two groups at T0 (ICC > 0.9).

### Comparison of volumetric measurements at T0 – T1 throughout the sample

Table 2 lists total airway volume and minimum airway cross-section at T0 and T1. Increases in NCTAV, NCMCS, NPMCS, HPTAV and HPMCS were observed, but the differences were not statistically significant. However, statistically significant increases were observed between T0 and T1 for OPTAV (*p* < 0.01), OPMCS (*p* < 0.05) and NPTAV (*p* < 0.05).

**Table 2:** Comparative analysis of nasal cavity, nasopharynx, oropharynx and hypopharynx at T0, T1, T0-T1

	T0 M (SD)	T1 M (SD)	T0-T1 M (SD)	*P* value	d Cohen
NCTAV (cm^3^)	17.23 (7.00)	18.95 (6.58)	-1.71 (7.42)	0.215	0.23
NCMCS (mm^2^)	157.58 (116.94)	166.55 (125.36)	-8.91 (120.14)	0.685	0.07
NPTAV (cm^3^)	4.20 (2.18)	4.78 (2.13)	-0.59 (1.42)	0.031*	0.41
NPMCS (mm^2^)	24.82 (27.27)	39.65 (59.55)	-14.83 (53.65)	0.141	0.27
OPTAV (cm^3^)	14.11 (5.85)	17.94 (7.81)	-3.83 (7.53)	0.009**	0.50
OPMCS (mm^2^)	137.53 (108.32)	190.77 (116.31)	-53.23 (126.46)	0.028*	0.42
HPTAV (cm^3^)	1.28 (0.67)	1.65 (1.37)	-0.37 (1.40)	0.157	0.26
HPMCS (mm^2^)	177.33 (168.32)	203.46 (101.97)	-26.12 (198.50)	0.477	0.13

*NCTAV* nasal cavity total airway volume, *NCMCS* nasal cavity minimal cross-section, *NPTAV* nasopharynx total airway volume, *NPMCS* nasopharynx minimal cross-section, *OPTAV* oropharynx total airway volume, *OPMCS* oropharynx minimal cross-section, *HPTAV* hypopharynx total airway volume and *HPMCS* hypopharynx minimal cross-section

^*^p < 0.05; **p < 0.01

### Comparison of volumetric measurements at T0-T1 in patients treated with MARPE and BAME

Table 3 lists the measurements obtained at T0 and T1 for patients treated with MARPE. An increase was found in NCTAV, NCMCS, NPTAV, NPMCS, OPTAV, OPMCS and HPTAV, but none of the differences were statistically significant.

**Table 3:** Comparative analysis of nasal cavity, nasopharynx, oropharynx and hypopharynx at T0, T1, T0-T1 in MARPE

	T0 M (SD)	T1 M (SD)	T0-T1 M (SD)	*P* value	d Cohen
NCTAV (cm^3^)	17.03 (6.81)	20.07 (5.72)	-3.03 (7.45)	0.137	0.40
NCMCS (mm^2^)	142.58 (93.78)	194.64 (133.19)	-52.05 (132.91)	0.152	0.39
NPTAV (cm^3^)	4.00 (2.47)	4.16 (2.18)	-0.16 (1.46)	0.678	0.10
NPMCS (mm^2^)	32.54 (35.68)	45.37 (69.96)	-12.83 (63.40)	0.446	0.20
OPTAV (cm^3^)	13.41 (5.92)	15.07 (6.04)	-1.66 (5.63)	0.272	0.29
OPMCS (mm^2^)	126.91 (120.18)	154.27 (107.71)	-27.35 (107)	0.339	0.25
HPTAV (cm^3^)	1.46 (0.66)	1.92 (1.81)	-0.46 (1.81)	0.335	0.25
HPMCS (mm^2^)	193.66 (228.50)	186.04 (113.02)	7.62 (266.67)	0.913	0.02

*NCTAV* nasal cavity total airway volume, *NCMCS* nasal cavity minimal cross-section, *NPTAV* nasopharynx total airway volume, *NPMCS* nasopharynx minimal cross-section, *OPTAV* oropharynx total airway volume, *OPMCS* oropharynx minimal cross-section, *HPTAV* hypopharynx total airway volume and *HPMCS* hypopharynx minimal cross-section

Table 4 lists the measurements obtained from the BAME-treated patients at T0 and T1. An increase that was not statistically significant was observed in all study variables; the exception was NCMCS, which decreased.

**Table 4:** Comparative analysis nasal cavity, nasopharynx, oropharynx and hypopharynx at T0, T1, T0-T1 and *p*-value in BAME

	T0 M (SD)	T1 M (SD)	T0-T1 M (SD)	*P* value	d Cohen
NCTAV (cm^3^)	17.43 (7.42)	17.83 (7.38)	-0.40 (7.41)	0.837	0.05
NCMCS (mm^2^)	172.57 (138.01)	139.47 (114.54)	34.10 (90.85)	0.168	0.37
NPTAV (cm^3^)	4.40 (1.91)	5.41 (1.95)	-1.01 (1.27)	0.008**	0.79
NPMCS (mm^2^)	17.11 (11.82)	33.94 (48.82)	-16.83 (43.97)	0.160	0.38
OPTAV (cm^3^)	14.81 (5.91)	20.81 (8.49)	-6.01 (8.69)	0.018*	0.69
OPMCS (mm^2^)	148.15 (98.09)	227.27 (116.47)	-79.12 (142.28)	0.049*	0.55
HPTAV (cm^3^)	1.11 (0.66)	1.38 (0.68)	-0.27 (.89)	0.247	0.31
HPMCS (mm^2^)	160.99 (76.84)	220.87 (90.07)	-59.87 (89.79)	0.022*	0.66

*NCTAV* nasal cavity total airway volume, *NCMCS* nasal cavity minimal cross-section, *NPTAV* nasopharynx total airway volume, *NPMCS* nasopharynx minimal cross-section, *OPTAV* oropharynx total airway volume, *OPMCS* oropharynx minimal cross-section, *HPTAV* hypopharynx total airway volume and *HPMCS* hypopharynx minimal cross-section

^*^p < 0.05, ^**^p < 0.01

Table 5 compares data from patients treated with MARPE and BAME for T0-T1. For both the MARPE and BAME therapies, there was an increase in all of the indicators except for two: the minimum cross section of the hypopharynx for MARPE, which decreased slightly, and the minimum cross section of the nasal cavity for BAME, which also decreased. We found differences between MARPE and BAME at T0-T1 in terms of the minimum cross section of the nasal cavity, which increased for MARPE (52.05 ± 132.91 mm^2^) and decreased for BAME (-34.10 ± 90.85 mm^2^).

**Table 5:** Difference between T0 and T1 with MARPE and BAME

	MARPE T0-T1 M (SD)	BAME T0-T1 M (SD)	*P* value	d Cohen
NCTAV (cm^3^)	-3.03 (7.45)	-0.40 (7.41)	0.340	0.35
NCMCS (mm^2^)	-52.05 (132.91)	34.10 (90.85)	0.048*	0.75
NPTAV (cm^3^)	-0.16 (1.46)	-1.01 (1.27)	0.100	0.62
NPMCS (mm^2^)	-12.83 (63.40)	-16.83 (43.97)	0.842	0.07
OPTAV (cm^3^)	-1.66 (5.63)	-6.01 (8.69)	0.118	0.59
OPMCS (mm^2^)	-27.35 (107)	-79.12 (142.28)	0.270	0.41
HPTAV (cm^3^)	-0.46 (1.81)	-0.27 (.89)	0.722	0.13
HPMCS (mm^2^)	7.62 (266.67)	-59.87 (89.79)	0.361	0.33

*NCTAV* nasal cavity total airway volume, *NCMCS* nasal cavity minimal cross-section, *NPTAV* nasopharynx total airway volume, *NPMCS* nasopharynx minimal cross-section, *OPTAV* oropharynx total airway volume, *OPMCS* oropharynx minimal cross-section, *HPTAV* hypopharynx total airway volume and *HPMCS* hypopharynx minimal cross-section

^*^p < 0.05

## Discussion

MARPE and BAME are microimplant-assisted separators techniques indicated in cases of transverse maxillary deficiency. The primary adverse effect associated with MARPE is the buccolingual angulation (BLA) of the posterior teeth [35]. Additionally, increased molar torque has been linked to bone dehiscence with MARPE [36]. CBCT studies have revealed alterations in molar BLA following treatment with either MARPE or BAME [17, 24, 35, 37, 38]. Other factors, such as root resorption of the first molars, have been examined in prior studies [39], and root resorption has been revealed in teeth not directly linked to the appliance. Nevertheless, cases treated with skeletal-supported appliances exhibited reduced levels of resorption [40].

The objectives of this study were to investigate the volumetric and minimal section changes of the upper airway of patients with maxillary compression treated with MARPE and BAME. The different measurements were taken in the CBCT carried out before (T0) and after (T1) the maxillary expansion therapy. Other methodologies have been described in the literature, including upper airway aerodynamics [41] and superimposition with semi-automatic software [42]. Lo Giudice et al. [42] compared 20 CBCT records by superimposition with semi-automatic software and obtained proper results in terms of accuracy and efficiency.

The volumetric changes that we recorded in patients treated with MARPE were larger than those obtained in other studies [11, 43, 44]. Li et al. [10] performed a MARPE therapy on 22 patients and also obtained a volumetric increase of the nasal cavity of 2.92 ± 4.97 cm^3^. That increase was slightly smaller than what we obtained (3.03 ± 7.45 cm^3^). We noted a significant increase in the size of the nasal cavity and nasopharynx observed one and a half months after MARPE and BAME. However, further research is required to examine the enduring stability of these changes. It was not possible to compare the volumetric changes in the remaining topographic areas of the upper airway between both studies because different anatomical boundaries were used.

The volumetric changes that we recorded in the nasopharynx for both MARPE- and BAME-treated cases exhibited a slight increase. That finding is consistent with the published literature [10, 43–46]. Kim et al. [43] performed MARPE therapy on 14 adult patients and also obtained a volumetric increase of 0.64 ± 0.82 cm^3^ at the level the nasopharynx. That increase is slightly larger than what we obtained (0.16 ± 1.46 cm^3^). Unfortunately, the anatomical boundaries that Kim et al. used differed slightly from ours. Variations in the volumetric changes between these two studies may be attributed to changes in the placement location of the four micro-implants; they were more posterior in our study.

We recorded changes at the oropharynx level of 1.66 ± 5.63 cm^3^. That is consistent with the findings of previous studies [44, 45]. However, Yi et al. [46] reported a decrease of 1.08 ± 5.47 cm^3^ in a sample of 19 young adult patients who underwent BAME. But it is important to note that this change was not statistically significant. The activation rate used by Yi et al. was much slower than the one we used.

An increase in the hypopharynx was also observed following both MARPE and BAME therapies, but the difference was not statistically significant. Tang et al. [45] performed MARPE on 30 adult patients and performed CBCT prior to the expansion and after 3 months of retention. These authors recorded a volumetric decrease at the level of the hypopharynx. However, that study was carried out in a different way than ours; Tang et al. placed the anatomical limit between the hypopharynx and oropharynx at the tip of the epiglottis. Our results revealed a statistically significant increase in air volume at the nasopharynx and oropharynx level in cases treated with BAME.

This study does have limitations. First of all, our sample size was small and was characterized by a wide spread of ages. Also it is necessary to take into consideration that sample groups are different. Second, our observational period was relatively short (1.5 months); taking measurements after device removal may be useful to corroborate our findings. Additionally, changes in airway could have been studied using dynamic measures such as spirometry, peak expiratory and inspiratory flow or computational fluid dynamics. In addition, there was possible bias that derived from positioning the patients’ heads during the X-rays.

The results of this study have important implications for orthodontists. Microimplant-assisted separation devices can yield increases in the upper airway. However, there do not appear to be any statistically significant differences between MARPE and BAME therapies in terms of airway improvement; both therapies yielded similar results (with the exception of NCMCS).

## Conclusions

- An increase in total area and minimal section at the level of nasopharynx and oropharynx was observed in cases treated with microimplant-assisted expansion.
- No differences were found between MARPE and BAME treatments in terms of total airway volume and minimal section of the nasal cavity; the exception was NCMCS, which increased for MARPE and decreased for BAME.

### Supplementary Information

**Additional file 1:** **Fig. 1.** CBCT measurement of the nasal cavity in T0. **Fig. 2.** CBCT measurement of the nasopharynx in T0. **Fig. 3.** CBCT measurement of the oropharynx in T0. **Fig. 4.** CBCT measurement of the hypopharynx in T0. **Fig. 5.** CBCT measurement of the nasal cavity in T1. **Fig. 6.** CBCT measurement of the nasopharynx in T1. **Fig. 7.** CBCT measurement of the oropharynx in T1. **Fig. 8.** CBCT measurement of the hypopharynx in T1.

## Data Availability

All of the material is owned by the authors and/or no permissions are required. The datasets generated during and analyzed during the current study are not publicly available due to [national data protection law] but are available from the corresponding author on reasonable request.

## Abbreviations

RPE: Rapid Palatal Expansion
SARPE: Surgical-Assisted Rapid Palatal Expansion
MARPE: Microimplant-Assisted Rapid Palatal Expansion
BAME: Bone-Anchored Maxillary Expansion
CBCT: Cone-Beam Computed Tomography
NCTAV: Nasal Cavity Total Air Volume
NCMCS: Nasal Cavity Minimum Cross-Section
NPTAC: Nasopharynx Total Air Volume
NPMCS: Nasopharynx Nasal Cavity Minimum Cross-Section
OPTAC: Oropharynx Total Air Volume
OPMCS: Oropharynx Nasal Cavity Minimum Cross-Section
HPTAV: Hypopharynx Total Air Volume
HPMCS: Hypopharynx Nasal Cavity Minimum Cross-Section
ICC: Intraclass Correlation Coeficieny
ME: Measurement Error
