# Supplementary material for: Short-term outcomes in the upper airway with tooth-bone-borne vs bone-borne rapid maxillary expanders
Source: BMC Oral Health. 2023 Oct 4;23:714. doi: 10.1186/s12903-023-03461-6 (PMC10552363; doi:10.1186/s12903-023-03461-6)
Supplement: Supplementary file 1 — Additional file 1: Fig. 1. CBCT measurement of the nasal cavity in T0. Fig. 2. CBCT measurement of the nasopharynx in T0. Fig. 3. CBCT measurement of the oropharynx in T0. Fig. 4. CBCT measurement of the hypopharynx in T0. Fig. 5. CBCT measurement of the nasal cavity in T1. Fig. 6. CBCT measurement of the nasopharynx in T1. Fig. 7. CBCT measurement of the oropharynx in T1. Fig. 8. CBCT measurement of the hypopharynx in T1. [file 12903_2023_3461_MOESM1_ESM.docx]

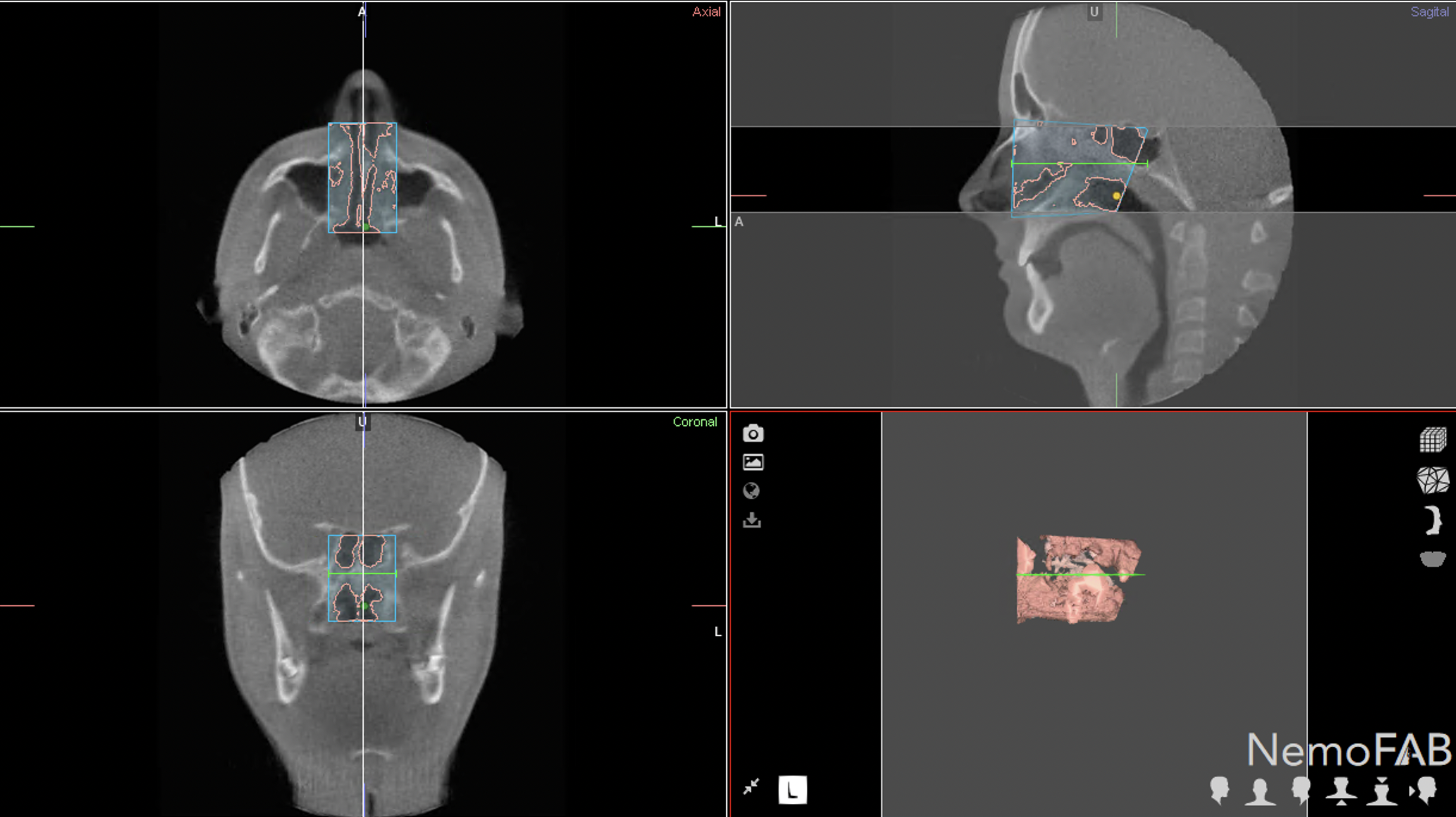


Fig. 1 CBCT measurement of the nasal cavity in T0


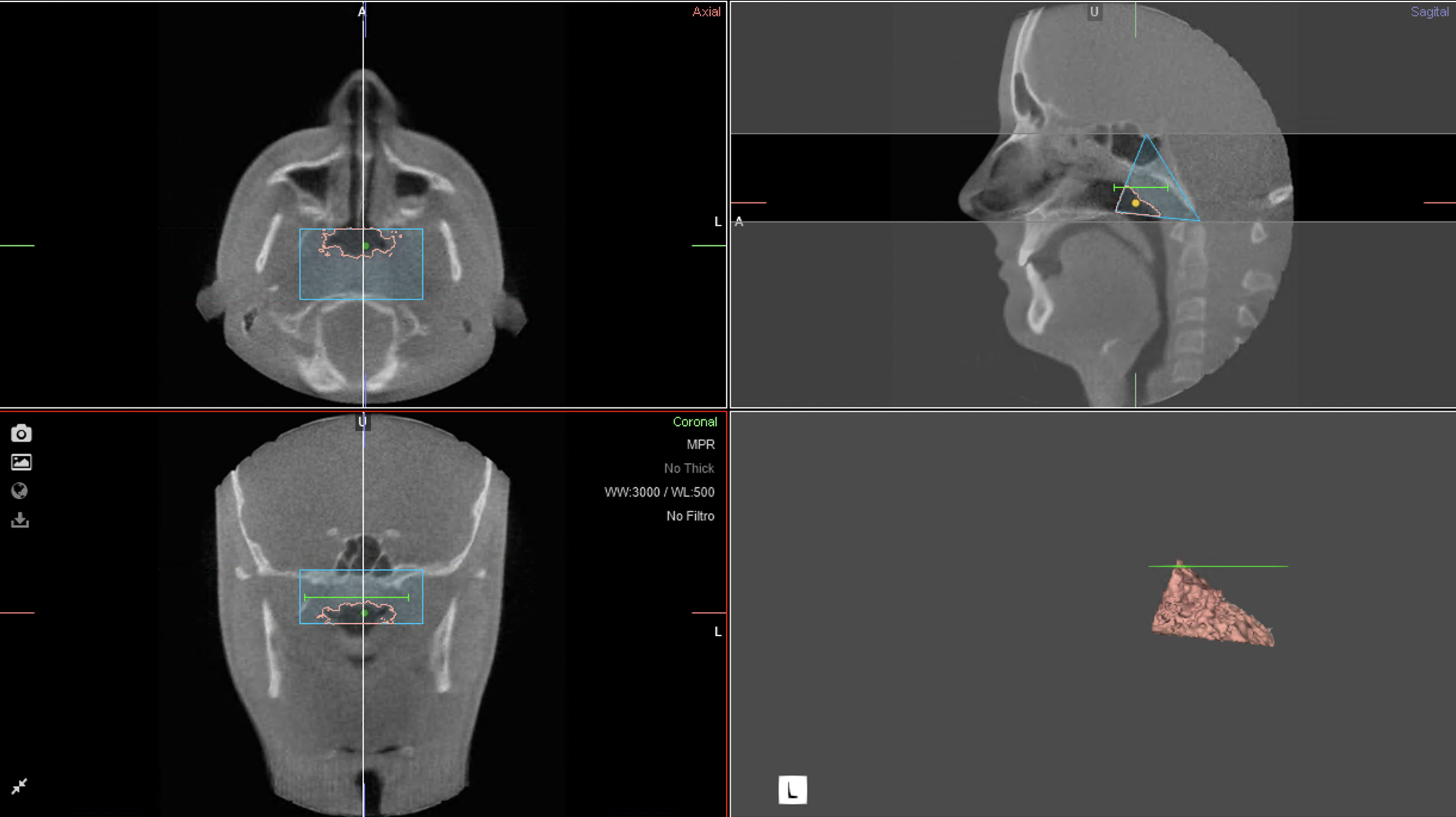


Fig. 2 CBCT measurement of the nasopharynx in T0


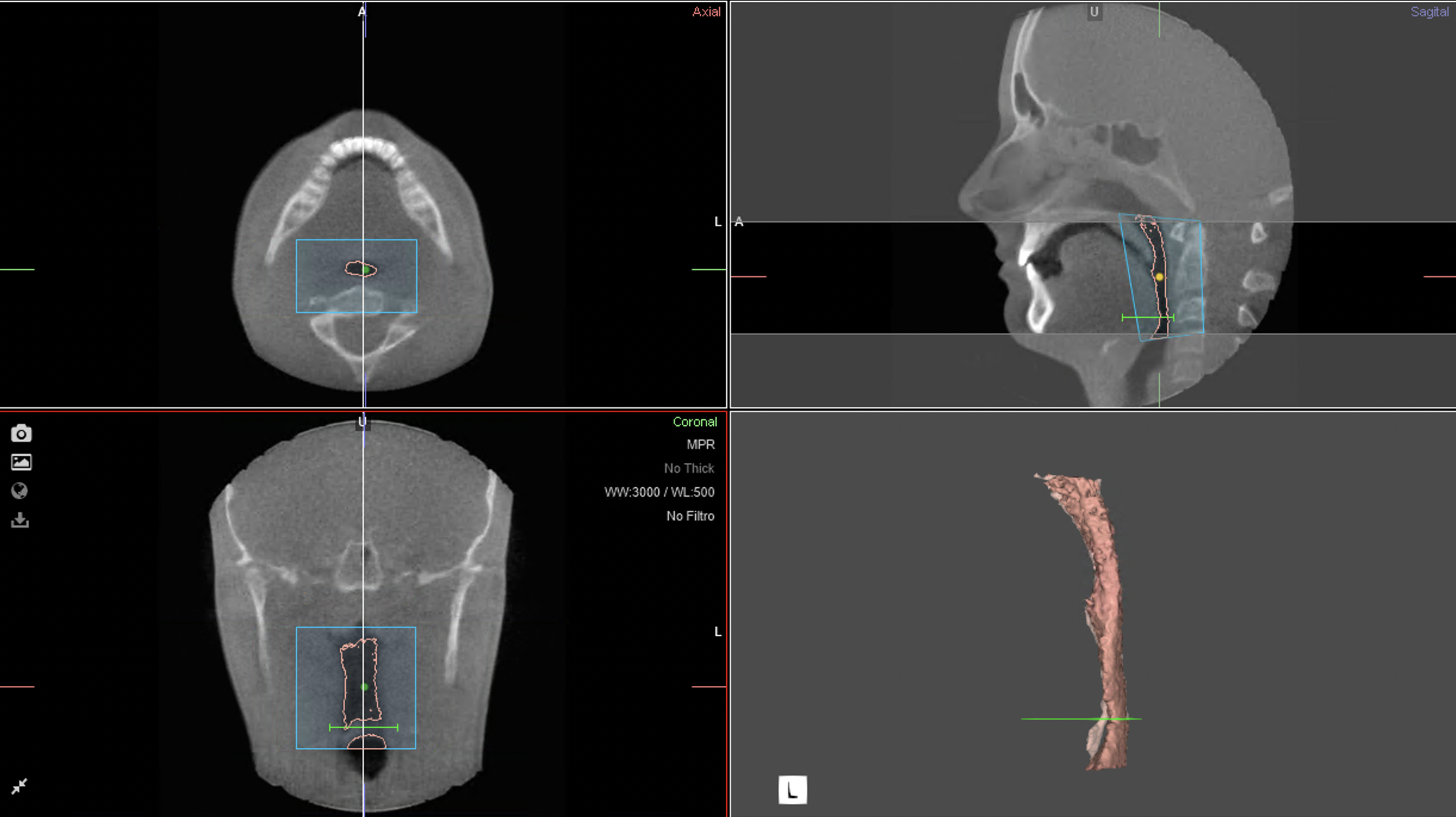


Fig. 3 CBCT measurement of the oropharynx in T0


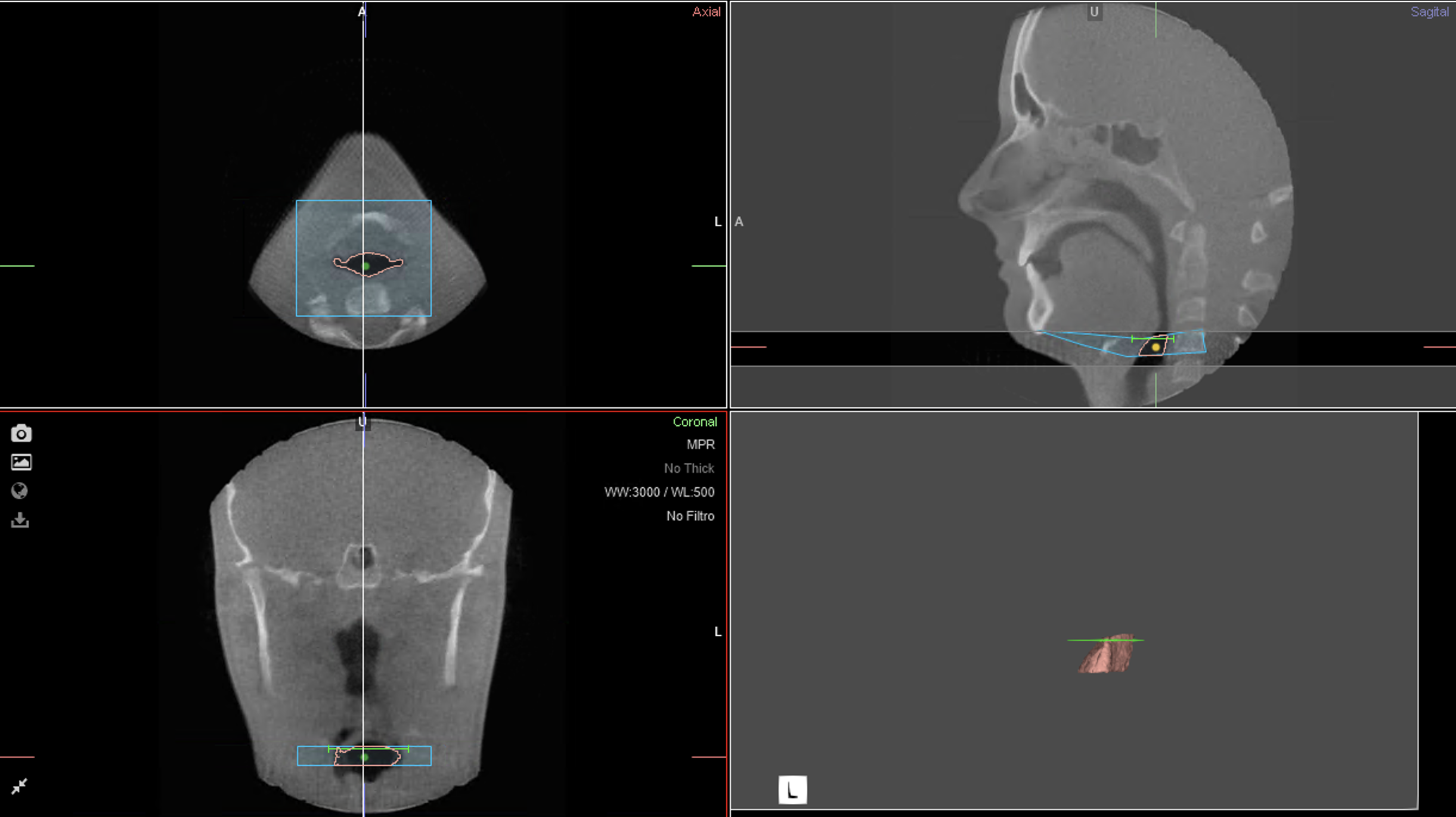


Fig. 4 CBCT measurement of the hypopharynx in T0


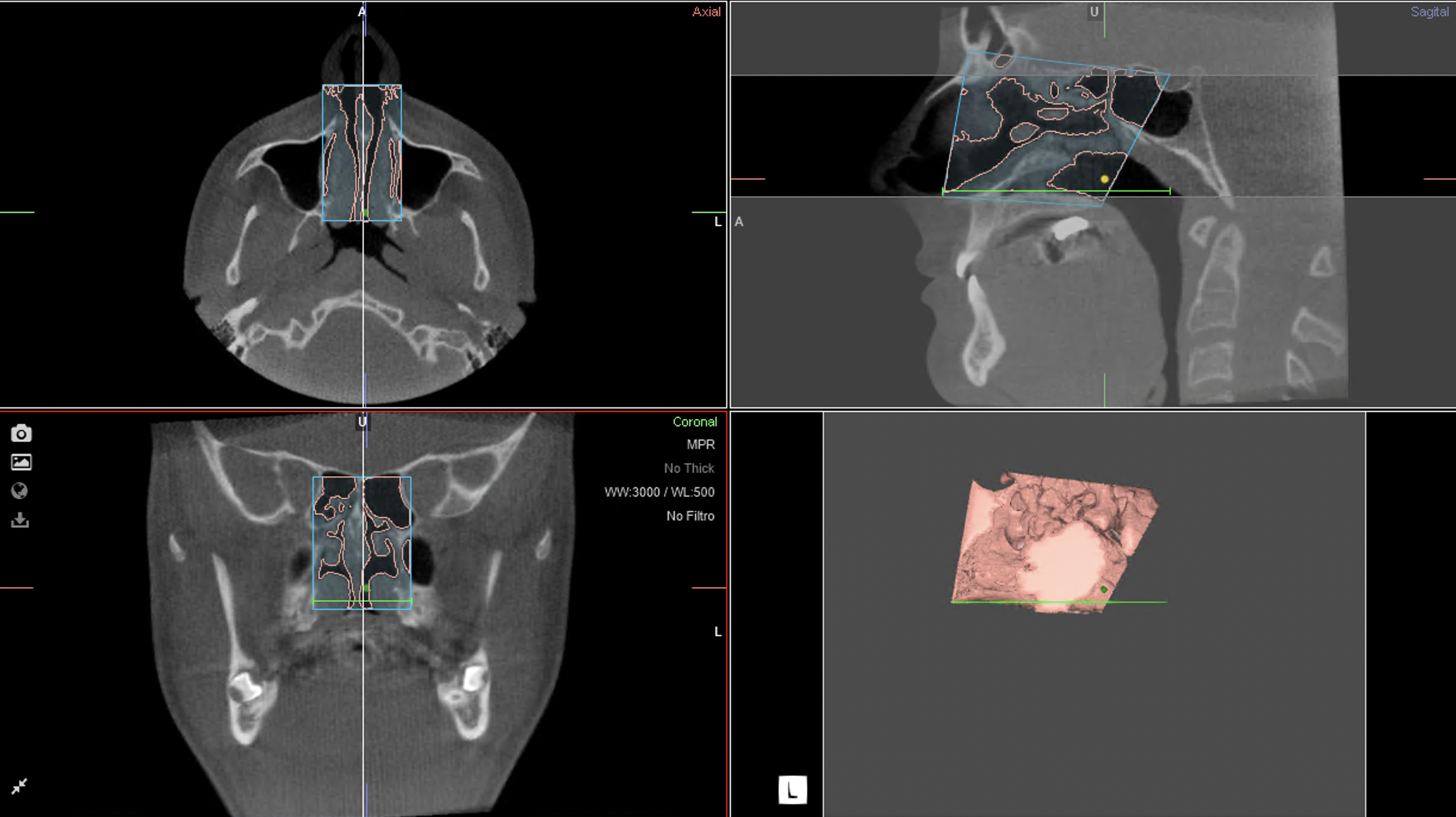


Fig. 5 CBCT measurement of the nasal cavity in T1


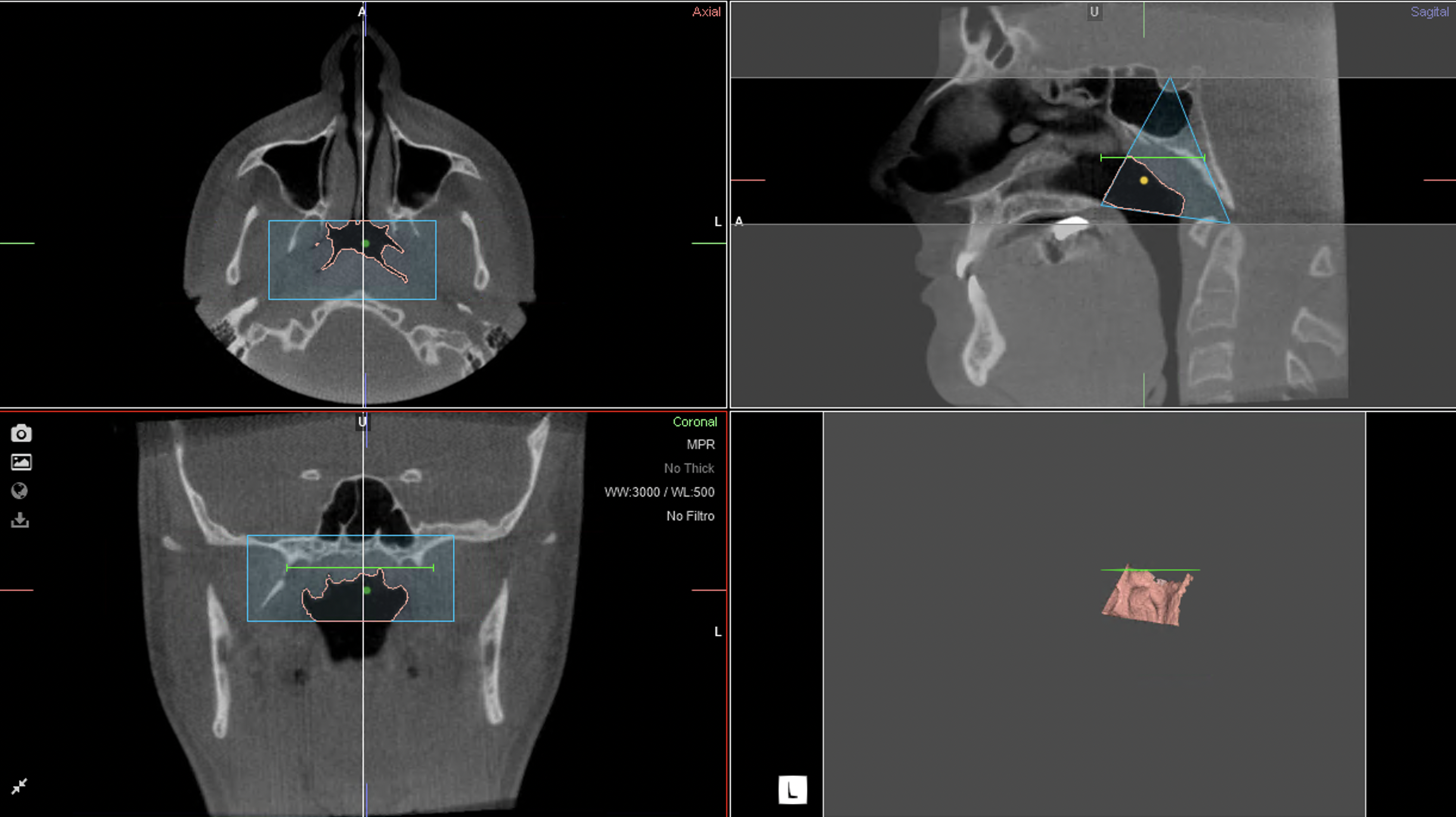


Fig. 6 CBCT measurement of the nasopharynx in T1


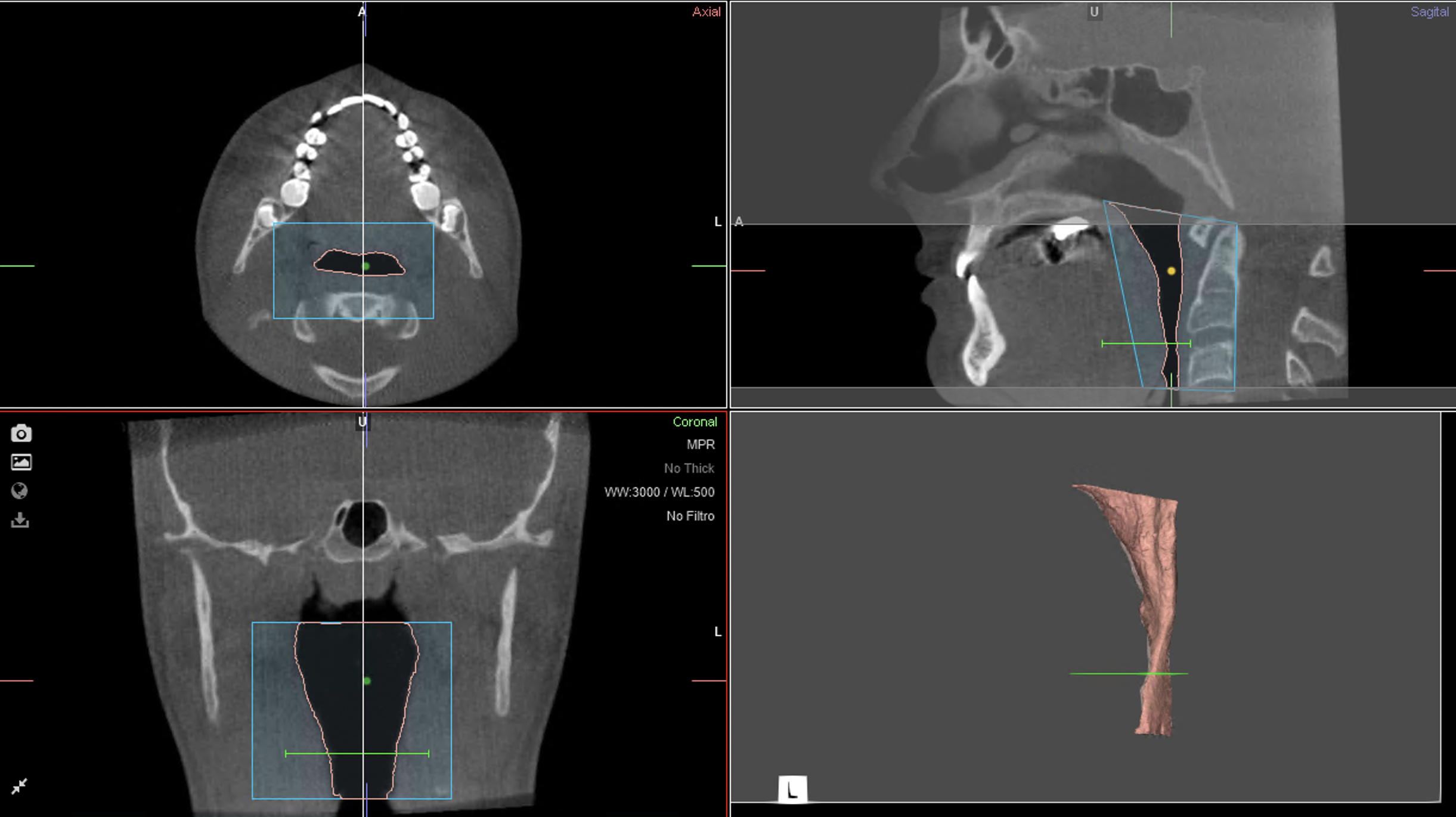


Fig. 7 CBCT measurement of the oropharynx in T1


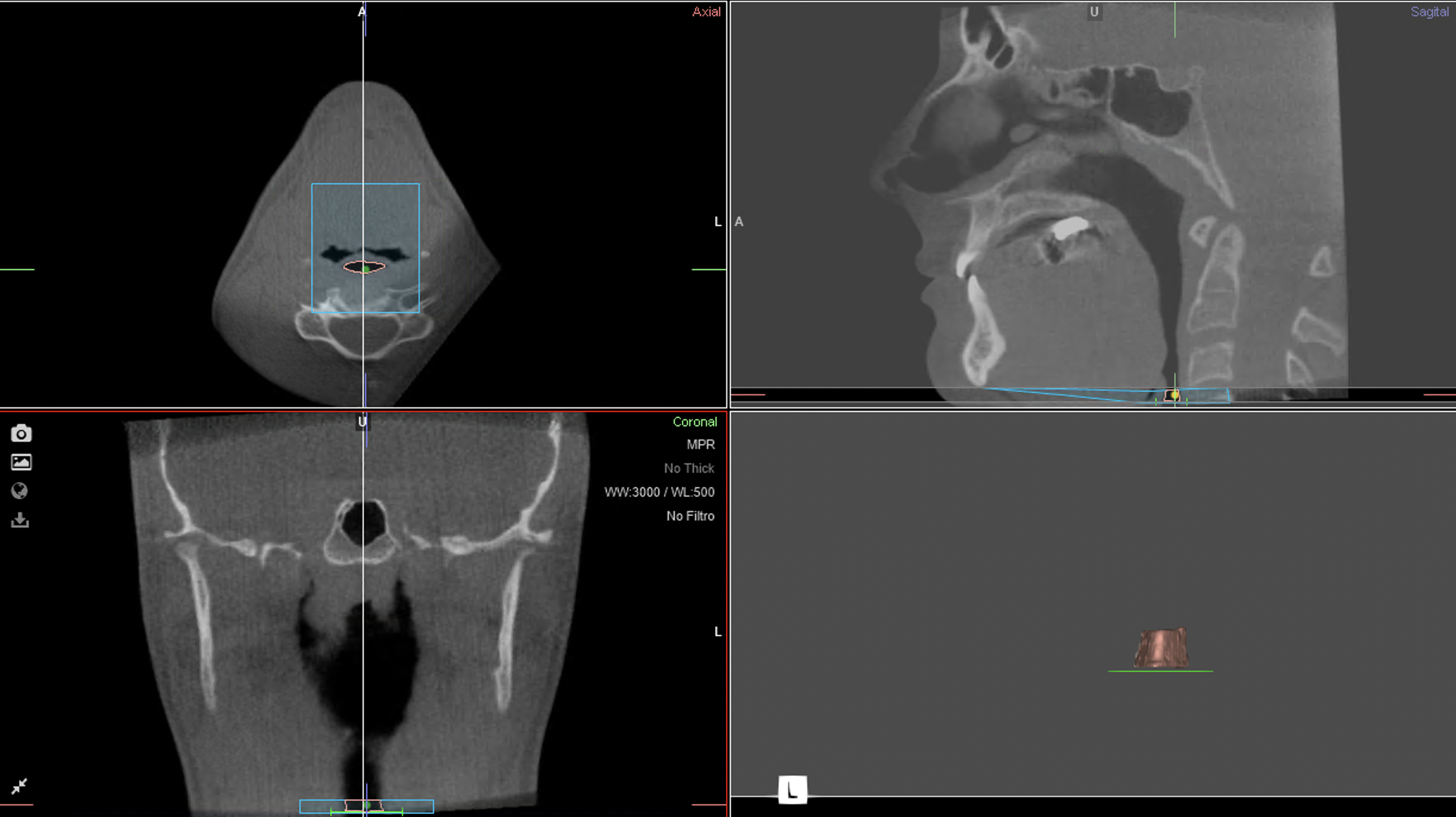


Fig. 8 CBCT measurement of the hypopharynx in T1
